# Capability and opportunity in hot shooting performance: Evidence from top-scoring NBA leaders

**DOI:** 10.1371/journal.pone.0179154

**Published:** 2018-02-12

**Authors:** Shun-Chuan Chang

**Affiliations:** Holistic Education Center, Mackay Medical College, New Taipei City, Taiwan; Universita degli Studi di Verona, ITALY

## Abstract

In basketball games, whenever players successfully shoot in streaks, they are expected to demonstrate heightened performance for a stretch of time. Streak shooting in basketball has been debated for more than three decades, but most studies have provided little significant statistical evidence and have labeled random subjective judgments the “hot hand fallacy.” To obtain a broader perspective of the hot hand phenomenon and its accompanying influences on the court, this study uses field goal records and optical tracking data from the official NBA database for the entire 2015–2016 season to analyze top-scoring leaders’ shooting performances. We first reflect on the meaning of “hot hand” and the “Matthew effect” in actual basketball competition. Second, this study employs statistical models to integrate three different shooting perspectives (field goal percentage, points scored, and attempts). This study’s findings shed new light not only on the existence or nonexistence of streaks, but on the roles of capability and opportunity in NBA hot shooting. Furthermore, we show how hot shooting performances resulting from capability and opportunity lead to actual differences for teams.

People still remember Ty Cobb, who stole 96 bases out of 134 tries (70 percent).

But they have forgotten Max Carey, who stole 51 bases out of 53 (96 percent).

-Positioning: The Battle for Your Mind (1981).

## Introduction

Since 1970, the National Basketball Association’s (NBA’s) scoring title is awarded to the player with the most points per game. Among the most recent winners, Kevin Durant has won four scoring titles, and Stephen Curry only one, in the 2015–16 season. However, in these seasons, their teams are not likely to win any NBA championship. Wouldn’t a team with both two top-tier scorers, Durant and Curry, become a basketball superpower? For another instance, a starting lineup of five All-Stars in the 2012–13 Los Angeles Lakers season still did not sufficiently improve the franchise to contend for the NBA championship.

Since basketball is a team sport, winning cannot be based only on one player’s—even an explosive scorer’s—contribution. In NBA history, except for only one player, Michael Jordan (six times), having a scoring winner rarely entails a final championship for the scorer’s team. Outstanding performance by an athlete can universally influence perceptions among players, coaches, and fans, creating an exciting atmosphere for almost everyone in the arena and outside, especially when the sophisticated player’s shot-by-shot performances can be broadcast live. In psychology literature, basketball players have been described as “having a hot hand” if they have a prolonged series of substantially good performances. Gilovich, Vallone, and Tversky [1] defined a “hot hand” in basketball as the belief that during a particular period, a player’s performance is significantly better than expected based on his overall record. Gilovich et al. [1] also found that 91% of fans agreed that a player has a better chance of successfully making a shot after having just made his last two or three shots. The conventional definition of hot hand, used in numerous studies [1, 2, 3], is the following:

Pr(Hit|Hit)>Pr(Hit|Miss). (1)

In other words, when a previous attempt is successful, the probability that the next attempt will be successful is higher than if the previous attempt was unsuccessful. Thus, the mathematical definition, based on such a micro view of competition, focuses on the probability of success between successive attempts in a relatively short period of time. This study, however, investigates whether this definition can be extended to longer periods, for instance, from shot-by-shot to game-by-game. For example, at the start of the 2015–2016 season, the Golden State Warriors set a record by winning 24 games in a row. The point guard Stephen Curry, who was also the NBA’s Most Valuable Player for that season, had an astonishing, on-fire performance in the season’s first week. In the season opener at the end of October, Curry scored 40 points; at the beginning of November, he scored 53 points. People worldwide became enamored with his skill at sinking difficult three-point shots. *Sports Illustrated*.*com* and the NBA’s official website on October 28th, 2015, used expressions such as “Check out all 24 of Stephen Curry’s first quarter points,” “Stephen Curry explodes for 40, but no other Warrior scored more than 13 points,” or “Curry with fresh legs, a hot hand and in top form is pretty great.”

Because of the high point total, reporters are commonly re-emphasizing perceived good performance in terms of large numbers, and most people would intuitively consider Curry to have hot hands, similar to the perceptions of Ty Cobb’s stolen bases in the epigraph. In other words, his missing one or more shots was not considered to indicate that a hot shooting streak had broken, so the overall popular perception persists.

In addition to the tendency for more successful shots to draw much more attention, some might also think that better players would make a higher percentage of the field goal they attempt, but this is generally not the case. Consider Curry’s shooting percentage statistics: almost 0.53 in the opening game. Curry’s making 14 out of 26 shots would not have been a big surprise, but if he had missed 12 in a row and then made 14 in a row, or if his coach had benched him after missing ten shots in a row, denying him any opportunity for a comeback, this immediately raises the question of traditional definition of hot hand. Although human behaviors and activities can be observed within a social context, unlike coin tosses and other objective, pure tests of probability, much of the basketball research on streaks has limited on fixed/controlled conditions, such as free throw shooting, three-point contests [2, 4], or designed experiments in which subjects made all their attempts from the same location [5].

These possible scenarios lead us to search for a broader perspective of the hot hand phenomenon and its accompanying influences on the court. Furthermore, shooting performance really includes field goal percentage, points scored, and attempts. The interplay of these three key shooting variables conducted in a strict analysis remained relatively unexplored. Our main research purpose is to employ statistical models to integrate these different shooting perspectives to explore the meaning of successful shots. In our study, we used the actual shooting records to analyze NBA top-scoring leaders’ shooting performances. The results may always be applicable to real world situations, and offer a more meaningful context for formulating specific competitive and cooperative strategies in a basketball game, where players’ behavior and choices naturally occur.

### Feeding the hot hand

Gigerenzer and Todd [6] focused on adaptive thinking in which humans and animals make decisions with limited time, knowledge, and computation. In basketball, Gilovich et al. [1] indicated to fans and players that passing the ball to someone who had just made several (two or more) shots was a common decision when aiming for more scoring. However, a multitude of conditions can be found on the basketball court. Snap shooting decisions made by a single player differ each time based on factors such as where the shot is taken, distance from the basket, and distance from defenders. In addition, players with hot hands, who are more willing to take shots because of increased confidence, are more likely to be heavily guarded by the opposing team [2]. These conditions on the court can produce confounding variables that substantially affect real performance outcomes. One noted study that attempted to resolve the issue of confounding factors was presented at the MIT Sloan Sports Analytic Conference by Bocskocsky et al. [7]. Data from the NBA’s optical tracking system in the 2012–2013 season revealed that a player with hot hands faced tighter defense, was more likely to receive the ball from teammates, and was more willing to attempt more difficult shots. These authors constructed a regression model to assess the likelihood of taking a shot. After controlling for the difficulty of each shot, they found evidence for a small, but significant hot hand effect.

On the other hand, just because many psychological studies have described the hot hand fallacy and the gambler’s fallacy as arising from subjective randomness (as compared to objective probability models) and determined that the perception of hot hands reflects common cognitive or memory biases (streaks are more memorable) and misperception of chance [8, 9], many studies have depicted the subjective belief in streaks or distorted evaluation of facts as primarily the effect of the law of small numbers [10, 11, 12]. In other words, characteristics and patterns of performance that are assumed or subjectively perceived to exist in long sequences are also expected to be observable in short sequences, and vice versa.

Burns [13] re-analyzed field goal data from the 48 home games of the Philadelphia 76ers during the 1980–81 season (seen in Table 1[1]), to calculate each player’s proportion of total number of shots comprising parts of runs of 1, 2, or 3 hits or misses. Burns [13] showed correlations between a player’s shooting percentage and the proportion of his shots. All runs of misses were highly negatively correlated with shooting percentage, while all runs of hits were highly positively correlated with shooting percentage (all p < .01). This supports the argument that streaks are predictive or a proxy of a player’s shooting percentage. In other words, coaches and players believe that, if a player has a hot hand in shots, they should “feed the hot hand” by giving him the ball more often, as a heuristic signal of a generally higher field goal percentage.

**Table 1:** Probability of making a hot game conditioned on the outcome of previous games.

								Serial
Player	P(hot│3 colds)	P(hot│2 colds)	P(hot│1 cold)	P(hot)	P(hot│1 hots)	P(hot│2 hots)	P(hot│3 hots)	correlation
Stephen Curry	0.58	0.48	0.49	0.43	0.32	0.27	0.33	-0.08
James Harden	0.56	0.53	0.55	0.49	0.43	0.29	0.40	-0.03
Kevin Durant	0.58	0.45	0.48	0.42	0.33	0.20	0.00	-0.08
DeMarcus Cousins	0.55	0.45	0.44	0.45	0.45	0.31	0.25	0.03
LeBron James	0.43	0.56	0.57	0.51	0.44	0.35	0.33	0.08
Damian Lillard	0.38	0.58	0.51	0.48	0.44	0.56	0.56	-0.11
Anthony Davis	1.00	0.78	0.68	0.54	0.45	0.53	0.50	-0.16
Russell Westbrook	0.60	0.47	0.53	0.50	0.48	0.42	0.38	-0.13
DeMar DeRozan	0.36	0.50	0.48	0.46	0.47	0.35	0.17	-0.19
Paul George	0.57	0.59	0.58	0.51	0.44	0.33	0.17	-0.11
Isaiah Thomas	0.83	0.65	0.55	0.54	0.52	0.48	0.45	-0.03
Klay Thompson	0.58	0.50	0.47	0.44	0.43	0.33	0.00	-0.16

Note: all p > .05.

### Field goal attempts as the Matthew effect in allocation of shots

Although the controversy surrounding the hot hand phenomenon has always existed, Hales [14] approached this problem from an epistemological perspective, which shifted the focus to individual differences. Hales [14] suggested that if basketball legend Michael Jordan made 10 free throws in a row, then Jordan himself would think he had hot hands. Hales made some intriguing arguments: Any better-than-average performance can be considered hot hands, players usually know when they are performing better than usual, and spectators can usually tell when a player is performing better than usual. All these viewpoints shed light on the hot hand phenomenon. Getting to the root of the problem of hot shooting performance requires developing statistical definitions better suited to actual conditions on the basketball court.

An explosive scorer’s franchise still struggles to gain the NBA championship, similar to the example of Durant and Curry in the previous section of this article. This is reminiscent the paradox of sophisticated skills: Teams that have been called “single-super-star” teams or even “selfish-leader” teams may show signs of the Matthew effect [15, 16], the term coined by Robert K. Merton [16] to originally describe how eminent scientists will often get more credit than a comparatively unknown researcher, and such a concept is also applicable to matters of fame or status, cumulative advantage of economic capital or expertise. Matthew effect is similar to the old Chinese saying “People always reduce scarcity and pay tribute to surplus,” from Laozi’s Tao Te Ching. Partly due to feeding the hot hand, we assume that the Matthew effect will be seen among basketball players. That is, the more-skilled players perform better and control more opportunities to shoot, and others on the same team with limited field goal attempts may perform worse than expected.

Underlying the Matthew effect in sociology is the assumption that has long posited status to be an indicator of hierarchical position and prestige that helps individuals and organizations procure resources and opportunities for advancement [15]. We further reflect on the relationship of the “hot hand” and “Matthew effect” in actual basketball competition. Since a basketball team is thus like a society’s economic system, the bad relationship between “hot hand” and “Matthew effect” is that super-star scorers may sometimes neglect their teammates, resulting in their teams failing to function cohesively. In other words, if all of one team’s field goal attempts per game are almost a constant, the critically hard part is slicing the pie and ensuring each teammate performs well when sharing the one ball.

Notably, the traditional field goal percentage is a key indicator for assessing a player’s capacity to score points. The equation for field goal percentage is field goals made per game divided by field goal attempts per game. If a player’s point total is bolstered purely by attempts made, then the increased number of attempts may actually harm team performance. The NBA tracks a more comprehensive statistic, true shooting percentage (TS%), which is calculated by points scored divided by two times the sum of field goals attempted and 0.44 times free throws attempted. The introduction of the TS% has revealed that many team managers have begun to focus on the practical implications of a more comprehensive interpretation of field goal percentage because no team wants an indiscriminate shooter. However, can the TS% accurately measure a player’s effectiveness in turning attempts into points? In recent years, for sharpshooters whose scoring comes mostly from three-pointers, we find this indicator has a complex explanation if it exceeds 100% in some games. Therefore, exploring how to measure successful shots derived from a player’s true shooting capability becomes the most critical issue in this study.

### Successful shoots as a function of capability and opportunity

In summary of many studies [14, 17, 18], when a basketball player is said to be on a streak according to the law of small numbers and the representativeness heuristic, three separate conditions are implied: (1) When a player’s field goal percentage is higher than usual, the next attempt is likely to be successful; conversely, when a player has cold hands, his or her field goal percentage will continue to be lower than usual. (2) When a player is performing well, he will score more points than usual; conversely, when a player is performing poorly, he will score fewer points than usual. (3) The performance, whether better or worse than usual, is relatively consistent over a period of time. To obtain a broader perspective of the hot hand phenomenon and its accompanying influences on the court, we assume that a scorer j tries to make his field goals attempted (FGA_j_) as similar to field goals made (FGM_j_) or achieved as possible. Specifically, we assume that the player aims to minimize the expected squared difference between the actual outcome and attempts, E(FGA_j_- FGM_j_)^2^ = (1/n) Ʃ _i_ (FGA_ji_- FGM_ji_)^2^, for each game i, given one player j, known as mean square error for player j, MSE_j_. FGA_ji_−FGM_ji_ can be written as the sum of FGA_ji_—E(FGA_j_) and E(FGA_j_)- FGM _ji_.

A good scorer j is one with a lower MSE derived from all his playing games, which comes from E(FGA_j_)- FGM _ji_ as capability, and FGA_ji_—E(FGA_j_), as opportunity. Here capability means using one’s average attempts effectively to achieve the specific field goals per game. Opportunity refers to aggregate events assisted by other teammates or circumstances that are advantageous to gain field goal attempts. In an analogy to MSE that incorporates both the variance of the estimator and its squared bias in decision theory, we assume that (FGA_ji_- FGM_ji_)^2^ = *f* (E(FGA_j_)- FGM_ji_, FGA_ji_—E(FGA_j_)), for each game i, given one player j, as the following Eq (2), which incorporates observable factors and captures the heterogeneity of individual players’ shooting skill:

Successfulshootingperformanceinagame=f(Capability,Opportunity). (2)

## Empirical illustrations

### Research data

This study included optical tracking data and traditional offensive performances in basketball from each game of the 2015–2016 season, reported on the official NBA website (http://stats.nba.com/). Although in baseball or basketball, a player's contract year season may deviate positively from long-term performance, we chose top-scoring NBA leaders (given by points scored per game) as our research subjects. The leading scorers are not only relevant samples for analysis of a hot shooting performance, but also have relatively consistent performance in recent years, for example, for the top 12 scorers in the 2015–2016 season, 9 of them were on the Top14 ranking list in the 2014–2015 season. In the league, they are typically acknowledged to be the best offensive players in the game, the stars having many fans and the benefit of the Matthew effect in allocation of shots, and almost all are among the most highly paid players in NBA.

From this valuable novel data point, optical tracking data, also called SportVU data, developed by the company responsible for the player tracking process (SportsVU, Northbrook, IL, USA), were obtained from six high-speed motion capture cameras installed in every basketball arena’s rafters. This system captures all movements, including positions of players and referees, and most importantly, the ball on the court, converting them into data. The official NBA website compiles several contingency tables of player statistics after data manipulation (http://stats.nba.com/tracking/-!/player/). Unlike traditional characteristics, optical tracking accurately captures court dynamics and presents a game’s process. For example, optical tracking can examine shooting efficiency variables: catch and shoot, pull-up shots, drives, and close shots, as well as touching and passing variables (i.e., setting up an assist for another teammate, called “secondary assists”) [19].

In addition, the official NBA website publishes key traditional statistics that are also incorporated into this study. These include basic game-by-game information such as FGA, field goal percentage, and points scored (listed separately for two-point and three-point field goals). More importantly, the official website provides statistics in the score box for each player, such as rebounds, assists, steals, blocks, turnovers, personal fouls, and +/- (a metric that looks at how teams perform with a certain player on or off the court). These data, combined with recent player tracking technology, provided us more information for understanding and observing changes that a player made on the court, particularly in subtle changes of defensive and offensive stability across games, to understand how players adjust to overcome performance slumps.

#### Study 1: Game-by-game analysis of conditional probabilities

Gilovich et al. [1] posed a primary question: whether the probability of successfully making a field goal is higher if a player had successfully made a field goal in the preceding attempt than if the player had been unsuccessful. By comparing the nine major players of the Philadelphia 76ers during the 1980–1981 season, the original study found the probability of only one player scoring a shot after a previously successful shot was higher than the probability of scoring after missing the previous shot. However, the serial coefficients were negative for eight of the nine players. In other words, the authors confirmed that the hot hand phenomenon for shot-by-shot performance was a fallacy. In our proposed study, we used each game as the basis for analyzing the sample of 12 top-scoring leaders in the 2015–2016 season, as described in Table 1. We also performed a conditional probability analysis. For each player, we defined a game as hot or good if the player’s field goal percentage in that game was greater or equal to his average field goal percentage in the 2015–2016 season; all other games were defined as cold or bad. From this game-by-game analysis, we tested the existence of hot and cold shooting performance among games, throughout a full NBA season, based on a probability interpretation.

In contrast to the analysis between shots in Gilovich et al. [1], the serial correlations in Table 1, column 9 were almost negative for 10 of the 12 players, but no coefficients significantly differed from zero; this means that for each game throughout the season, these top scorers’ field goal percentage (FG%) performances were consistent or random, rather than streaky or skewed: comparisons of column 7, P (hot/2 hots) with column 3, P (hot/2 colds), of column 8, P (hot/3 hots) with column 2, P (hot/3 colds), and column 6 with column 4 as well, (paired t = 7.86, p < .01 for columns 7 and 3; t = 4.46, p < .01 for columns 8 and 2; and t = 4.61, p < .01 for columns 6 and 4). For instance, only one player (Lillard), out of the 12 players, had a P(hot/3 hots) greater than his P(hot/3 colds), and some players’ P(hot/3 hots) were even null. This provided additional evidence against the streak-shooting performance associated with game-by-game analyses. Psychologist Daniel Kahneman, a Nobel Award winner in economics, offered a possible explanation for the kind of hot hand cognitive illusion that occurs when we are attempting game-by-game analysis throughout the season: Substantial increases in FG% performance are naturally followed by decreases in performance (showing negative serial correlations). This is also called the concept of regression toward the mean [20].

Additionally, to test whether hot shooting performance predictions are associated with those in a previous game, we further estimated the following OLS regression for each top scorer: *y_i,j_* = *β*_0,*j*_ + *β*_1_*y*_*i*−1,*j*_ + *ε*_*i*,*j*_. Here *y*_*i*,*j*_ is the shooting performance indicator in the *i*th game, including field goal percentage, points scored, and attempts made by player *j*. Each *R*^2^ for these regressions was below 0.04, suggesting that this causal model did not explain a substantial part of the variance in predictions by the lagged dependent variable. All players had a value |${\hat{\beta }}_{1}$| < 0.2 (all p > .10), which was consistent with analytical evidence against streak-shooting performance under different shooting perspectives. We further checked the residuals of these regression lines regarding normality, homoscedascticity, and independence. After using the Kolmogorov-Smirnov (K-S) test for normality, Bartlett test for homoscedascticity, and Durbin-Watson (D-W) test for independence, we found that these hypothesis testing results were all shown non-significant (all p > .10), meaning that the error term assumptions of OLS regression were met.

#### Study 2: Analysis of runs

Gilovich et al. [1] defined a run as a sequence of field goals or a sequence of missed baskets. They then performed the Wald-Wolfowitz Runs Test and predicted that if the hot hand phenomenon for shot-by-shot was real, the number of runs should be fewer than expected if results were based on pure chance. We defined each game as an analytical unit and considered sequences of hot games (H) or cold games (C) that we defined as a run. In this context the null hypothesis was that Hs and Cs would be randomly distributed within the sequence, so the past history of the sequence would not influence the chance that the next game would be H or C. An alternative hypothesis was that the past history of the sequence would have some effect on whether the next game is H or C. For example, we observed there were three runs in the five-game series HHCCH.

In Table 2, a comparison of columns 5 and 6 indicates that the expected number of runs were actually greater than the actual number of runs for only two of the players. The z statistic reported in column 7 shows no significant difference at the .05 level between the observed and expected number of runs. A z-score is also known as a standard score. That is, z-scores range from -3 standard deviations (which would fall to the far left of the normal distribution curve) up to +3 standard deviations (which would fall to the far right of the normal distribution curve). Thus, we conclude that as a whole, these top scorers do not reflect significant streakiness or hot hand shooting behavior in field goal percentage (FG%) performance throughout the season.

**Table 2:** Runs test.

				Number	Expected				
				of	number of			average hot length	average cold length
Number	Player	Hots	Colds	runs	runs	Z	P-value		
1	Stephen Curry	34	45	45	39.73	1.22	0.22	1.55	1.96
2	James Harden	40	42	46	41.98	0.89	0.37	1.74	1.83
3	Kevin Durant	30	42	40	36.00	0.98	0.33	1.50	2.10
4	DeMarcus Cousins	29	36	32	33.12	-0.28	0.78	1.81	2.25
5	LeBron James	39	37	43	38.97	0.93	0.35	1.77	1.76
6	Damian Lillard	36	39	40	38.44	0.36	0.72	1.80	1.95
7	Anthony Davis	33	28	37	31.30	1.48	0.14	1.83	1.47
8	Russell Westbrook	40	40	42	41.00	0.23	0.82	1.90	1.90
9	DeMar DeRozan	36	42	39	39.77	-0.18	0.86	1.89	2.10
10	Paul George	41	40	46	41.49	1.01	0.31	1.78	1.74
11	Isaiah Thomas	44	38	42	41.78	0.05	0.96	2.10	1.81
12	Klay Thompson	35	45	41	40.38	0.14	0.89	1.75	2.14
	**Mean**	**35.8**	**39.1**	**41.00**				**1.76**	**1.91**

Note: all p > .05.

Furthermore, Albert et al. [21] suggested that runs of longer length form a stronger impression and are more likely to be recognized as the hot hand phenomenon. In addition to the law of small numbers, there is the theory of selective attention [13] which states that when people receive large amounts of information, they selectively focus on the information that easily matches their interests and experiences. Sun and Wang [22] also proposed the phenomenon of waiting time, in which a rare streak pattern attracts attention.

Thus, although the run test above can assess a run’s randomness, it cannot ascertain its length. To supplement this, we added two new columns (9, 10) to indicate the average length of hot runs and cold runs, respectively, and to verify the differences between the two runs. In particular, the average length of hot runs was less than the average length of cold runs, but non-significant (paired t = −1.506, p = .16).

#### Study 3: Analysis of successful shooting performance

In Table 3, all results are based on the standardized OLS regressions from the pooled top 15 scorers’ data. We pooled across game-by-game data for each game i and player j, and found that their average points per game were greater than 20. Each E(FGA_j_) was calculated from all games clustered on individual player j. That is, E(FGA_j_) = (1/n) Ʃ _i_ FGA_ji_, for each game i, given one player j. Thus, our model as Eq (2) suggests that (un)successful shooting performance can be accounted for by two independent variables: (1) X1, opportunity, FGA_ji_—E(FGA_j_) and (2) X2, capability, E (FGA_j_)- FGM_ji_. The standardized regression has been a common tool for assessing the effect and predictive or explanative power of each independent variable [23], especially for only two independent variables. The standardized regression coefficient here has been used to assess the relative importance of opportunity and capability.

**Table 3:** The standardized regressions results from pooled top scorers data.

	(1) All data		(2) PTS≧30		(3) PTS≦15	
Variable	Coefficient	p-value	Coefficient	p-value	Coefficient	p-value
			**(a)FG**			
X1	1.348	<0.01	1.218	<0.01	1.180	<0.01
X2	0.908	<0.01	0.785	<0.01	0.672	<0.01
N	1132		284		142	
ΔR^2^	0.430		0.397		0.310	
R^2^	0.948		0.959		0.957	
			**(b) 3-Point FG**			
X1	1.003	<0.01	1.010	<0.01	0.887	<0.01
X2	0.879	<0.01	0.820	<0.01	0.953	<0.01
N	1132		284		142	
ΔR^2^	0.576		0.521		0.696	
R^2^	0.889		0.909		0.879	

As in Table 3, column (1), which includes all data (N = 1132 games), two independent variables are significant. For both FG and 3-point FG, two standardized regression coefficients of X1 and X2 in the modeling results implied that opportunity is relatively more important than capability, accounting for general shooting performance. However, in the case of 3-point field goals, ΔR^2^ = 0.576, showing that the coefficient of partial determination can be defined as the additional proportion of variation explained by the player’s capability (X2) specified in a regression model including both regressor X1 and X2, but cannot be explained by opportunity (X1) when considering only X1 in a regression model. The result of ΔR^2^ from the part of (b) 3-point FG also shows more explanatory power for capability in our regression model after controlling for the effect of opportunity (X1), compared to results from the part of (a) FG.

Column (2) in Table 3 shows the results when we used only data for which the scorers’ outcome in a game was above or equal to 30 (in about 25% of these games). Consistent with column (1), this implied that although opportunity was still more important than capability in hot shooting, long-distance shooting was based much more on capability or skill rather than luck, allocations to shoot, or assists from others; in short, opportunity. Column (3) in Table 3 documents the results when we used only data for which actual scorers’ outcomes were below or equal to 15 (about one-eighth of all games). Standardized regression coefficients were significant and positive. However, for 3-point field goals, capability was relatively more important than opportunity by a small amount. In fact, there were worse shooting performances, with limited 3-point field goal attempts making smaller scoring contributions.

We further calculated the MSE and several average shooting performance measurements for each player throughout the season. As seen in Table 4, MSE had a significant positive correlation with points (PTS) at the 5% level, but not with the true shooting percentage (TS%) nor with the three-point field goals percentage (3P%); MSE showed negative associations with field goal percentage (FG%) at the 5% level. All pairwise Pearson correlation coefficients were non-significant among points, field goal percentages, and three-point field goal percentages. True shooting percentage was significantly associated with three-point field goal percentages or field goal percentages at the 1% level, but MSE generally showed higher correlation coefficients with points (PTS) than with other indices.

**Table 4:** Pearson correlations between different shooting performance measurements among players.

	MSE	PTS	TS%	FG%
**MSE**				
**PTS**	.515*			
**TS%**	-0.356	0.513		
**FG%**	-.521*	0.326	.644**	
**3P%**	-0.356	0.074	.728**	0.214

**P < .01

*P < .05.

#### Study 4: Analysis of the Matthew effect in allocation of shots

To examine whether these 15 top-scoring NBA leaders emerged because outstanding points scored are strongly associated with opportunity, we estimated the following OLS regression for each top scorer: *y*_*i*,*j*_ = *β*_0,*j*_ + *β*_1_*X*_*i*,*j*_ + *ε*_*i*,*j*_. Here *y*_*i*,*j*_ represents points scored in *i*th game by player *j*, and *X*_*i*,*j*_ is the variable of opportunity, for the *i*th game observed by player *j*, that corresponds to X1 in Study 3. R_j_^2^ for these regressions ranged from 0.20 to 0.63 (another case of special data calculated from Kobe Bryant’s performance in his last NBA season in 2015–2016, interestingly R^2^ = 0.72), suggesting that this simple model can explain meaningful predictions of points scored by opportunity. Some players (8 of 15) had a value of ${\hat{\beta }}_{1}$ higher than 1.0, consistent with the fact that an explosive scorer may also miss too many field goal attempts, in other words, place too much weight on opportunity.

Moreover, each top scorer’s value of R_j_^2^ or ${\hat{\beta }}_{1}$ was negatively associated with the following indices from passing variables in NBA optical tracking data: passes made or received per game, free throw assists per game, secondary assists per game, potential assists per game, and points created by assist per game (the correlation is −0.02 to −0.39, but p-value > 0.05, two-tailed test). In addition, each top scorer’s value of R_j_^2^ or ${\hat{\beta }}_{1}$ was positively associated with the number of times a player touched and possessed the ball (correlation: 0.46 to 0.52, p-value < 0.05), consistent with our expectation that the more times a player touches or possesses the ball or the fewer the total number of passes he makes, the greater his scoring opportunities. We further checked the residuals of these regression lines regarding normality, homoscedascticity, and independence. After applying the Kolmogorov-Smirnov (K-S) test for normality, Bartlett test for homoscedascticity, and Durbin-Watson (D-W) test for independence, we also found that all these hypothesis testing results were non-significant (all p > .05), meaning that the error term assumptions of OLS regression were met.

The semi-partial correlations in Table 5 compare the unique variation of MSE (having removed variation associated with opportunity, named as adjusted MSE or MSE_adj_) with unfiltered variation of other statistics shown in the official box score for each game in our scoring leaders’ data. For example, with partial correlation, we find the correlation between MSE and one statistic shown in the official box, such as points, holding opportunity constant for both MSE and points. However we want to hold opportunity constant for only MSE, in that case, we compute a semi-partial correlation. A semi-partial correlation is computed between the residuals e from the simple linear regression, MSE = β opportunity + e, and another variable, such as points. Because we know that a game’s successful shooting performance means one with a low MSE, and after accounting for opportunity, MSE_adj_ in column (2) were significantly associated with all listed statistics (except blocks). Consistent with the statistics on field goal performance shown in columns (1) and (3), MSE_adj_ had a significant positive correlation with FGA but showed significant negative associations with field goal percentage, points scored, and team winning percentage (W/L). This implies that in this condition, assuming shooting opportunity as a constant, higher capability in shooting scores can reflect good performance, with fewer FGA for both two points and three points. Scorers with higher capability can also score more points and improve their teams’ probability of winning. The basketball plus-minus shown in the last row of Table 5 represents how teams perform with or without a certain player on the court as well as the overall strong impact that a top-scoring leader has on team success.

**Table 5:** Semi- partial correlations between the defensive, offensive and opportunity-adjusted MSEs across games.

	(1) PTS≧30		(2)30>PTS>15		(3) PTS≦15	
Variable	MSEadj		MSEadj		MSEadj	
	N = 284		N = 706		N = 142	
W/L	-0.148	*	-0.25	**	-0.313	**
points	-0.354	**	-0.319	**	-0.363	**
field goals made	-0.608	**	-0.57	**	-0.486	**
field goals attempted	0.14	*	0.203	**	0.246	**
field goals percentage	-0.78	**	-0.68	**	-0.64	**
3-point field goals made	-0.111		-0.088	*	-0.188	*
3-point field goals attempted	0.153	**	0.188	**	0.094	
3-point field goals percentage	-0.232	**	-0.197	**	-0.251	**
rebounds	0.081		0.107	**	0.084	
assists	0.122	*	0.132	**	0.029	
steals	-0.007		-0.08	*	0.013	
blocks	0.031		-0.002		0.001	
turnovers	0.194	**	0.214	**	0.088	
personal fouls	0.086		0.15	**	0.060	
Plus-Minus (+/-)	-0.297	**	-0.257	**	-0.382	**

**P < .01

*P < .05.

As seen in Table 5, columns (1) and (2), MSE_adj_ had a significant positive correlation with assists, showing that higher scoring capabilities reflect more for carrying determinant responsibility, but star (high-scoring) players tended not to pass the ball; however, their score performance sometimes slumped below 15, as shown in column (3). No significant differences between defense indices, such as rebounds, steals, and blocks, were found for any associations with MSE_adj_ in columns (1) and (3). In other words, during hot and cold shooting performances, these top scorers played a predominantly offensive, rather than defensive role. In addition, MSE_adj_ generally showed significant positive correlation coefficients with turnovers (except in column (3).

## Discussion

In summary, this study analyzed a regular NBA season using field goal records and optical tracking data. Results showed that if scorers pursued the scoring title for highest points per game, their shooting performance could be objectively evaluated in terms of covariates such as field goal percentage, points scored, and attempts. The results of our analysis of these individual variables in studies 1 and 2 both revealed consistent counterevidence to streak-shooting or hot hand performance from game-by-game analysis.

Statisticians Efron and Morris [24] collected data from 18 Major League Baseball players and verified that batting averages from the first 45 at-bats cannot accurately predict performance throughout the season. From a long-term perspective, final performance results should shrink toward the annual average regardless of how the season opens. Efron and Morris believe that development of a more accurate estimation method requires considering latent factors related to the situation and teammate relationships or chemistry. We believe this can also be explained through team-oriented work in basketball. In other words, the performances of each member of a team are correlated in some way, even if this correlation is not easily measured.

Assuming underlying field goal attempts as a reflection of the Matthew effect in allocation of shots, we used an available open dataset of top-scoring NBA leaders by ranking in our study. Of course, these data included all the best players with high reputations and expectations from evaluators. Thus, for example, we could properly examine points made by opportunity, (i.e., Curry, R^2^ = 0.51; Thompson, R^2^ = 0.58; Durant, R^2^ = 0.42), meaning that in these top-scoring leaders’ data, No. 12, Klay Thompson, needs more points made by opportunity than No. 3, Kevin Durant. In other words, when playing on the same team in the 2017 season, if Thompson cannot attempt as many shots as previously or fears failure and attempts only the sure shots allowed by his limited opportunities, he will at times experience lower scores in struggling situations. This will be especially so when the Matthew effect to find out who is the king of super stars in allocation of shots is occurring.

Constant practice makes perfect. Practice is undeniably required for all athletes to improve their capabilities and to excel on the field or court in competitive sports. However, is skill or ability the determining factor in a game? Statistical evidence from top-scoring NBA leaders in our study showed that, on most occasions, opportunity to shoot is more important than shooting capability for performance. We echoed Morrison and Kalwani [25], who examined National Football League (NFL) kicker statistics to ask whether these players are lucky or good and why certain kickers seem to perform better than others. But after reconfirming the latent heterogeneity of player skills with a beta-binominal distribution model, Morrison and Kalwani [25] actually found no significant differences in skills among these best NFL kickers, even after accounting for varied kicking distances. In this study, we applied arguments from decision theory that using MSE incorporates observed heterogeneity from game records to identify two discriminants, opportunity and capability. We derived these to obtain a more complete interpretation of explanative power, according to the weight of true capability in hot shooting performance among NBA scorers across a regular season’s games. This study found that separating opportunity and capability encourages better thinking about player outcomes and allows for sharply improved decision making to evaluate practice planning on the court.

## Conclusion

We have exploited real-field-setting data analysis involving numerous successful performances on the basketball court, correlated with and without adjusting scoring opportunity, to provide a clearer picture of the true quality of shooting capability and relevant performance indicators to explain how high-status scorers advanced to defensive and offensive playing over the long, 6-month regular season. Our findings may also help develop a logical way to identify which players truly facilitate success for others and which are more selfish. The initial examination of the data from the 2015–2016 season in our analysis of top-scoring leaders’ shooting performances might suggest a broader perspective of the hot hand phenomenon on the court, but more research is needed in this area. In particular, given that players play different numbers of games, a mixed model or hierarchical model could be considered. Although the research debate and discussion about the hot hand hypothesis are far from over, this study’s findings may renew interest not only in the existence or nonexistence of streaks, but in the roles of capability and opportunity in NBA hot shooting. Finally, from our modeling approach, we can recognize how to interpret the versatile performances of top scorers across the dimensions of their offense, defense, and passing in a professional basketball game.
